# A cross sectional study to compare cardiac structure and diastolic function in adolescents and young adults with youth-onset type 1 and type 2 diabetes: The SEARCH for Diabetes in Youth Study

**DOI:** 10.1186/s12933-021-01328-0

**Published:** 2021-07-07

**Authors:** Amy S. Shah, Scott Isom, Dana Dabelea, Ralph D’Agostino, Lawrence M. Dolan, Lynne Wagenknecht, Giuseppina Imperatore, Sharon Saydah, Angela D. Liese, Jean M. Lawrence, Cate Pihoker, Elaine M. Urbina, SEARCH for Diabetes in Youth Study Group

**Affiliations:** 1 Department of Pediatrics, Division of Endocrinology, Cincinnati Children’s Hospital Medical Center and The University of Cincinnati, 3333 Burnet Ave ML 7012, Cincinnati, OH 45229 USA; 2 Department of Biostatistics and Data Science, Wake Forest School of Medicine, Winston-Salem, USA; 3 Lifecourse Epidemiology of Adiposity and Diabetes (LEAD) Center, University of Colorado Anschutz Medical Campus (CU-Anschutz), Aurora, USA; 4 Division of Diabetes Translation, Centers for Disease Control and Prevention, Atlanta, USA; 5 Department of Epidemiology and Biostatistics, Arnold School of Public Health, University of South Carolina, Columbia, USA; 6 Department of Research & Evaluation, Kaiser Permanente Southern California, Los Angeles, USA; 7 Department of Pediatrics, The University of Washington, Washington, USA

**Keywords:** Type 1 diabetes, Type 2 diabetes, Cardiac structure, Diastolic function, Pediatrics, Young adults

## Abstract

**Aims:**

To compare left ventricular structure (LV) and diastolic function in young adults with youth- onset diabetes by type, determine the prevalence of abnormal diastolic function by diabetes type using published values from age similar healthy controls, and examine the risk factors associated with diastolic function.

**Methods:**

In a cross sectional analysis we compared LV structure and diastolic function from two dimensional echocardiogram in participants with type 1 (T1D) and type 2 diabetes (T2D) who participated in the SEARCH for Diabetes in Youth Study. Linear models were used to examine the risk factors associated with worse diastolic function.

**Results:**

Of 479 participants studied, 258 had T1D (mean age 21.2 ± 5.2 years, 60.5% non-Hispanic white, 53.9% female) and 221 had T2D (mean age 24.8 ± 4.3 years, 24.4% non-Hispanic white, 73.8% female). Median diabetes duration was 11.6 years. Participants with T2D had greater LV mass index and worse diastolic function that persisted after adjustment for differences in risk factors compared with participants with T1D (all p < 0.05). Abnormal diastolic function, quantified using healthy controls, was pronounced in both groups but greater in those with T2D than T1D (T2D: 57.7% vs T1D: 47.2%, respectively), p < 0.05. Risk factors associated with worse diastolic function included older age at diabetes diagnosis, female sex, higher BP, heart rate and HbA1c and longer diabetes duration.

**Conclusions:**

LV structure and diastolic function is worse in individuals with T2D compared to T1D. However, abnormal diastolic function in seen in both groups compared to published values from age similar healthy controls.

**Supplementary Information:**

The online version contains supplementary material available at 10.1186/s12933-021-01328-0.

## Introduction

The incidence and prevalence of youth-onset diabetes continues to rise [1, 2]. By 2050, it is anticipated nearly 600,000 youth will be impacted by type 1 diabetes and more than 80,000 will have type 2 diabetes in the U.S. alone [3]. Diabetes is associated with increased cardiovascular morbidity and mortality [4]. Particularly concerning for individuals with diabetes is the 2–fourfold higher risk of developing heart failure with preserved systolic ejection fraction, HFpEF [5, 6]. HFpEF currently affects 6.5 million adults and rates are increasing in large part due to the continued rise of diabetes [7]. Together, diabetes and HFpEF worsen patient outcomes, quality of life and increase health care costs [8]. Understanding whether youth-onset diabetes is associated with an increased risk of HFpEF is important given the potential impact on society.

Individuals with type 2 diabetes are at high risk for HFpEF due to diabetic cardiomyopathy, which is defined as abnormal cardiac structure and function in the absence of overt coronary artery disease. Diabetic cardiomyopathy leads to myocardial fibrosis and dysfunctional remodeling, including impaired left ventricular filling and left ventricular diastolic dysfunction [9]. Additional risk factors for diastolic dysfunction include obesity and hypertension [10]. There are no effective therapies for HFpEF, except prevention and early identification and treatment of the risk factors (obesity and hypertension) associated with worse diastolic function.

Prior work has demonstrated antecedents/risk factors of HFpEF particularly elevated left ventricular (LV) mass, lower diastolic function, and higher left atrial size are present in adolescents and young adults with type 2 diabetes higher relative to obese controls [11–13]. In separate studies of youth with type 1 diabetes, higher LV mass and worse diastolic function have been described [14–17]. Direct comparisons of left ventricular structure and diastolic function in participants with type 1 and type 2 diabetes are lacking, except for a small study of less than 20 adolescents with diabetes [18].

Thus, the objectives of this study were to 1) compare left ventricular structure and diastolic function in young adults with youth- onset diabetes by type, 2) examine the prevalence of abnormal diastolic function in young adults by diabetes type using published values from age similar healthy controls, and 3) examine the risk factors associated with worse diastolic function.

## Methods

### Description of the study participants

The SEARCH for Diabetes in Youth Study cohort is a longitudinal study of individuals with youth-onset (diagnosed < 20 years of age) type 1 or type 2 diabetes. The SEARCH cohort was recruited from the population-based SEARCH Registry that has ascertained youth-onset type 1 and type 2 diabetes cases from Colorado including Southwestern American Indian reservations, Ohio, Washington, South Carolina, and California continuously since 2002. Individuals in this analysis were diagnosed with type 1 or type 2 diabetes in 2002–2006 or 2008 and enrolled shortly after diagnosis (baseline). Follow-up visits were conducted in 2011–2015 and 2015–2019 among those with ≥ 5 years diabetes duration.

This is a cross sectional analysis of study participants who underwent an in-person study visit between 2015 and 2019 where an echocardiogram was performed. Four of the 5 SEARCH sites (Colorado, Ohio, Washington and South Carolina) conducted the echocardiogram assessments; it was not feasible to conduct echocardiograms at the California site. All participants with type 2 diabetes (N = 221) and the first participants until we reached enrollment target with type 1 diabetes were invited to an echocardiogram assessment. Comparison of participants with type 1 diabetes undergoing echocardiograms to the eligible sample of participants for a follow-up SEARCH visit revealed no significant differences in age, sex, race/ethnicity or hemoglobin A1c.

At the time of the echocardiogram assessment participants had a mean age ± SD of 22.9 ± 5.1 years old and a median (IQR) 11.6 (8.5; 13.3) years diabetes duration. Diabetes etiologic type was defined based on measures at the baseline visit [19]; individuals with clinically diagnosed diabetes that were insulin sensitive [20] or antibody positive were characterized as having type 1 diabetes (n = 226) and those that were insulin resistant [20] and islet cell antibody negative as having type 2 diabetes (N = 194). In 59 participants, either islet cell antibodies or insulin sensitivity measures were unavailable; for these participants, diabetes type was based on their provider diagnosis (32 with type 1, 27 with type 2 diabetes). Prior work in SEARCH has shown good agreement between etiologic and provider diagnosed diabetes [21] and for the n = 59 with provider diagnosed diabetes there were no differences in age, race, sex or hemoglobin A1c compared to those etiologic diagnosed diabetes.

### Informed consent

All participants or parent/guardians provided written informed consent and assent, as appropriate by age. Institutional review boards approval was obtained at each site.

### Data collection at the time of echocardiography

At the in-person visit anthropometric, demographic and metabolic variables were collected [22]. Medical history, current medications, sex and race were self-report. Current smoking status and physical activity were also self-reported and defined as cigarette smoking in the last 30 days and the number of days a participant performed “hard physical effort that made you breathe harder than normal for at least 10 min at a time”, respectively. Height and weight were measured and body mass index (BMI) was calculated as (kg/m^2^). BMI z scores were calculated using age and sex specific CDC growth charts. [23]. Blood was collected after a minimum of 8-h fast and all samples (lipids, hemoglobin (Hb) A1c) were analyzed at the Northwest Lipid Metabolism and Diabetes Research Laboratories at University of Washington, Seattle, Washington [24]. Details regarding assays have been reported [22]. Resting systolic blood pressure (BP) and diastolic BP were measured three times, using an aneroid sphygmomanometer and an appropriate-sized cuff, after the participants were seated for at least 5 min according to published guidelines [25]. BP z scores were calculated by comparing the observed BP to CDC growth charts which are age, gender, and height adjusted. For both BMI and BP z scores, if participants were older than age 20, age 19.999 reference data was used. Z scores were used in the final models as opposed to raw values given that the age range of the cohort was 10–36 years, z scores correct for difference in BMI and BP at different ages, and z-scores result in less bias in adults than raw values would result in adolescents.

### Echocardiography

Each site was trained to perform echocardiograms via webinar and all sonographers passed certification studies. In brief, a two-dimensional transthoracic echocardiogram was performed with the participant lying in a left lateral decubitus position to maximize image quality. Parasternal short axis, parasternal long axis, and apical 4 chamber views were obtained. This allowed measurement of the left atrial and left ventricular structure and left ventricular function including traditional and tissue Doppler imaging of mitral inflow. Echocardiograms were read by a single technician blinded to diabetes type at the central Echocardiography Reading Center (Cincinnati Children’s Hospital Medical Center) on Digiview (Digisonics, Houston, TX) for LA and LV structure and LV diastolic function. LV strain, stroke volume and ejection fraction were read on the TomTec system (Unterschleissheim, Germany). Coefficients of variation for LV structure and diastolic function measures were ≤ 5.7% with intraclass correlation coefficients ≥ 0.89 (unpublished data, 2020).

#### Cardiac structure and geometry

LV mass index mass (a measure of hypertrophy) and relative wall thickness (RWT) (a measure of LV wall thickness relative to LV cavity size) were calculated using indices of LV end-diastolic dimension, end-diastolic posterior wall thickness and end-diastolic septal thickness. LV mass was indexed by dividing LV mass by height in meters raised to 2.7 to minimise the effects of age, sex and race [26, 27]. Indexing to height was chosen over LVM /BSA as BSA may not account for severe obesity and has erroneously identified LVM as reduced in overweight adults [27]. Threshold levels for LV mass index and RWT were used to evaluate LV geometry. For LV mass index, an adult threshold of > 51 g/m^2.7^ was used because of its association with cardiac morbidity in adults [28]. For RWT, a common adult threshold of > 0.43 was used [29]. This resulted in four geometric categories: normal geometry, concentric remodelling (normal LVmass index and increased RWT), eccentric hypertrophy (increased LVmass index and normal RWT), and concentric hypertrophy (increased LVmass index and increased RWT). These categories of LV geometry are related to cardiovascular mortality, with concentric hypertrophy being the most severe, and are often a precursor to LV failure [30].

#### Cardiac function

LV systolic outcomes included shortening fraction, ejection fraction, strain and strain rate. Shortening faction and ejection fraction were assessed as previously described [11]. Global longitudinal strain from the four chamber view and global strain rate were obtained with tissue velocity imaging using a tissue Doppler technique. Diastolic function was assessed by the ratio of the transmitral peak early (E) and atrial (A) wave velocities. Lower E/A indicates mild diastolic dysfunction and elevated E/A severe, restrictive diastolic dysfunction [31].Tissue Doppler imaging provided velocity of relaxation of the myocardial wall at both the septal and lateral annuli and provided a corresponding early (e’) and atrial (a’) wave. The average of the septal and lateral e’ and a’ waves were used in analyses to calculate E/e’ (higher indicates diastolic dysfunction) and e’/a’ ratio (lower indicates diastolic dysfunction). These non-invasive measures of diastolic function correlate well with invasive measures of diastolic function (time constant of relaxation [tau]) and LV end-diastolic pressure [31].

To give a reference as to the extent of potential diastolic dysfunction in participants with type 1 diabetes and type 2 diabetes values for E/A, E/e’, and e’/a’ were compared to published values from age similar healthy controls (age range 18–30 years) [32, 33]. We defined abnormal diastolic function as values that were > or < than 2SD above the mean for healthy controls. As such abnormal diastolic function was defined as E/A < 0.7, E/A > 3, E/e' > 10, e'/a' < 1.5.

*Arterial Stiffness:* Pulse wave velocity (PWV) was assessed using the SphygmoCor CPV system (AtCor Medical, Lisle, IL) between the carotid and femoral artery (carotid femoral PWV) as previously described [34]. The average of at least 10 beats was used in the analysis to cover a complete respiratory cycle. Three PWV recordings were obtained per participant and averaged. A higher PWV indicating a *higher* arterial stiffness. Coefficients of variability were 7% for PWV.

### Statistical analyses

Data are presented as mean (SD) or median (IQR) for continuous variables, and count (%) for categorical variables. Comparisons of LV structure and function by diabetes type (type 2 diabetes v. type 1 diabetes) were evaluated using t-tests or Wilcoxon tests for continuous variables, and chi-square tests for categorical variables.

Linear models were used to examine the risk factors associated with worse diastolic function (outcomes). Variables were selected based on previous identified associations with diastolic function in youth [11, 12, 35, 36]. The initial model (Model 1) evaluated the association with the following baseline covariates: age at diagnosis, race/ethnicity, sex, diabetes type, diabetes duration, BP z score, lipids, HbA1c, physical activity, BMI z score and adjusted for clinical site. Subsequent models included baseline covariates but added either LVM index (Model 2) or PWV carotid femoral (Model 3) to determine whether risk factors associated with the diastolic outcomes were working through a stiffer heart (LVM index) or stiffer vessels (PWV carotid femoral), respectively. The final model (Model 4) included the baseline covariates + LVM index and carotid femoral pulse wave velocity. All outcomes are log transformed for modeling. Collinearity of covariates was checked using a condition index and variance inflation factor and no areas of concern were identified. All data was also re-examined using raw BMI and BP instead of z scores and though there were minor shifts in parameter estimates and p values, the overall results were unchanged.

Finally, a cluster analysis was conducted to analyze data from participants who had similar phenotypic characteristics. The number of clusters was determined using pseudo t-square and cubic clustering criteria measures as guides and then viewing the characteristics of the groups to identify meaningful similarities and differences. The association of the defined clusters with diastolic function was evaluated with linear regression models. All analyses were completed using SAS version 9.4 (SAS Institute Inc., Cary, NC).

## Results

Comparisons of demographic, anthropometric, and laboratory data of participants with type 1 and type 2 diabetes at the time of the echocardiography measurements are shown in Table 1. Type 1 diabetes participants were more likely to be Non-Hispanic white and male while type 2 diabetes participants were more likely to be Non-Hispanic Black and female. Despite similar duration of diabetes (by study design), participants with type 2 diabetes were older, had a worse CV risk profile (higher mean BMI, systolic and diastolic BP, HbA1c and worse lipids), and self-reported more smoking and less days of vigorous physical activity, all p < 0.05. More participants with type 2 diabetes vs type 1 diabetes were on statins (10.8% vs 4.8%, p = 0.02 respectively). There were no differences in the use of ACE inhibitors (13.4% vs 9.6%, p = 0.20 respectively) and angiotensin receptor blockers (3.6% vs 2.0%, p = 0.30 respectively) by type of diabetes.

**Table 1:** Participants characteristics by diabetes type at echocardiography visit, 2015–2019

	Type 1, n = 258	Type 2, n = 221	p value
Age (years)	21.2 ± 5.2	24.8 ± 4.3	< 0.0001
Sex, Female (n,%)	139 (53.9%)	163 (73.8%)	< 0.0001
Race			< 0.0001
Non-Hispanic White (n,%)	156 (60.5%)	54 (24.4%)	
Non-Hispanic Black (n,%)	48 (18.6%)	125 (56.6%)	
Hispanic (n,%)	29 (11.2%)	27 (12.2%)	
Asian, Native American (n,%)	25 (9.7%)	15 (6.8%)	
Diabetes duration (years)	11.9 (9.1; 13.3)	11.3 (6.8: 13.3)	0.1911
BMI (kg/m^2^)	25.9 ± 5.3	38.0 ± 9.5	< 0.0001
BMI z-score	0.8 ± 0.9	2.0 ± 0.6	< 0.0001
Total cholesterol (mg/dl)	172.5, 48.0	185.0, 58.0	0.0143
LDL-C (mg/dl)	103.4 ± 32.7	112.3 ± 40.0	0.0092
HDL-C (mg/dl)	56.3 ± 14.6	42.3 ± 11.6	< 0.0001
Triglycerides (mg/dl)	77.0 (55.5: 106.0)	122.0 (85.0: 193.0)	< 0.0001
Systolic BP (mmHg)	110.3 ± 12.0	123.2 ± 16.8	< 0.0001
Systolic BP z-score	− 0.5 ± 1.1	0.5 ± 1.2	< 0.0001
Diastolic BP (mmHg)	72.1 ± 10.3	80.1 ± 11.6	< 0.0001
Diastolic BP z-score	0.5 ± 1.0	1.2 ± 0.9	< 0.0001
Hemoglobin A1c (%)	9.0 ± 1.9	9.5 ± 3.0	0.0397
Current smokers (n%)	31 (12.3%)	42 (19.5%)	0.0319
Days of vigorous physical activity	2 (0; 4)	0 (0; 3)	0.0001

Data are mean ± SD, n (%) or median (IQR)

**Fig. 1:**
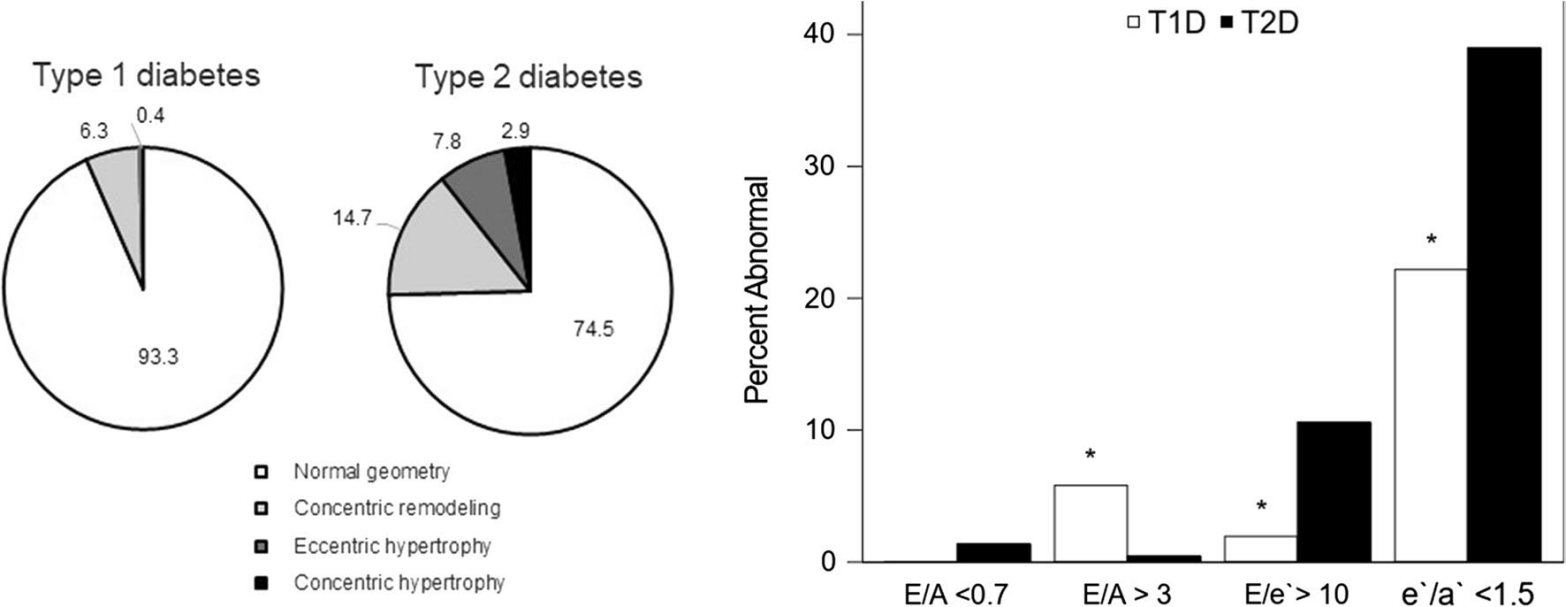
Left Ventricular Geometry and Diastolic Function by Diabetes Type. **A.** Distribution of Left Ventricular Geometry by Diabetes Type. Participants with type 1 and type 2 diabetes were stratified into four groups according to the LV mass cutoff > 51 g/m^2.7^ and relative wall thickness (RWT) cutoff of > 0.41: **A** Normal geometry (white), **b** concentric remodeling (increased RWT only, light gray), **c** eccentric hypertrophy (increased LVM only, dark gray), and **d** concentric hypertrophy (both increased LVM and RWT, black). p value difference between groups was < 0.0001. **B** Prevalence of Abnormal Diastolic Function by Diabetes Type. Diastolic function was compared to data from age similar healthy controls to assess the percent of abnormal diastolic function in participants with type 1 diabetes and type 2 diabetes [32, 33]

Data on cardiac structure, geometry and cardiac function by diabetes type are presented in Table 2. Unadjusted means showed higher LVM index and RWT in participants with type 2 diabetes compared to participants with type 1 diabetes (p < 0.05). After adjustment for BMI, systolic and diastolic BP, and HbA1c differences in LVM index persisted. There was also evidence of more abnormal cardiac geometry in participants with type 2 diabetes with a greater percent of participants having concentric remodeling, eccentric remodeling or concentric hypertrophy, p value difference between groups was < 0.0001, Fig. 1A.

**Table 2:** Cardiac structure and function from echocardiography by diabetes type

	Type 1, n = 258	Type 2, n = 221	Unadjusted p value
Cardiac structure			
LVM Index (g/m^2.7^), N: median(IQR)	29.2 (24.8: 33.4)	35.9 (30.4: 44.0)	< 0.0001*
LV relative wall thickness	0.3 (0.1)	0.4 (0.1)	< 0.0001
Left atrium length (cm) (4 chamber)/BSA	0.2 (0.0)	0.2 (0.0)	0.2536
Left atrium area (4 chamber)/BSA	0.7 (0.1)	0.7 (0.1)	0.4675
Cardiac function			
Shortening fraction (%)	36.0 ± 5.1	36.2 ± 5.9	0.7046
Ejection fraction (%)	57.2 ± 7.4	53.4 ± 7.5	< 0.0001*
Peak longitudinal strain (4 chamber)	− 20.6 ± 4.0	− 18.5 ± 3.5	< 0.0001*
Peak longitudinal strain rate (4 chamber)	− 1.0 ± 0.2	− 0.9 ± 0.2	< 0.0001*
E/A ratio	1.9 ± 0.5	1.6 ± 0.5	< 0.0001*
E/e' ratio	6.3 ± 1.5	7.6 ± 2.2	< 0.0001*
e'/a' ratio	1.9 ± 0.6	1.7 ± 0.5	< 0.0001
Pulse wave velocity m/s	5.9 ± 1.2	8.0 ± 1.7	< 0.0001*

Data are unadjusted mean ± SD, n (%) or median (IQR). Lower is worse except for E/e’ were higher value is worse. * indicates p < 0.05 remains after adjustment for BMI z-score, systolic BP z-score, diastolic BP z-score and hemoglobin A1c. LVM Index, E/A, E/e’, and e’/a’ ratios are log transformed for modeling. After adjustment p value for the difference in RWT was 0.13 and e’/a’ was 0.158

Participants with type 2 diabetes also had lower systolic ejection fraction and worse longitudinal strain and strain rate compared to those with type 1 diabetes (Table 2). Participants with type 2 diabetes also had worse LV diastolic function as evidence by lower E/A, e’/a’ and higher E/e’, all p < 0.05. The differences in E/A and E/e’ persisted after adjustment for BMI, systolic and diastolic BP and HbA1c. Pulse wave velocity was higher in participants with type 2 diabetes, before and after adjustment (< 0.0001) indicating greater arterial stiffness.

To gauge severity of diastolic function, we used threshold values from age similar healthy controls as a reference group (See Fig. 1B). The percent of any abnormal diastolic function was 47.5% in participants with type 1 diabetes and 58.1% in participants with type 2 diabetes (p value difference between groups was 0.0214).

Risk factors associated with a worse E/A were older age at diagnosis, higher heart rate and longer diabetes duration (Table 3) LVM index was not associated with E/A and adding LVM index to the model did not change the risk factors associated with E/A. PWV carotid femoral was associated with E/A, but adding it to the model did not change any the associations with age, heart rate or diabetes duration. Risk factors associated with a worse E/e’ were female sex and higher systolic BP z score. Type 2 diabetes status was marginally significant (p = 0.0570). BMI z was significant in the base model but when LVM was added, BMI z score was no longer significant. PWV carotid femoral was not associated with E/e’. Risk factors associated with worse e’/a’ were older age at diabetes diagnosis, higher heart rate, and longer duration of diabetes. As above, BMI z score was significant in the base model but no longer significant when either LVM index or PWV carotid femoral were added. In the final model, both LVM index and PWV carotid femoral remained significant.

**Table 3:** Risk factors associated with diastolic function in participants with type 1 and type 2 diabetes

Parameter	E/A (lower is worse)			E/e’ (higher is worse)			e’/a’(lower is worse)		
	Estimate	SE	p value	Estimate	SE	p value	Estimate	SE	p value
Intercept	1.919	0.156	< .0001	1.458	0.158	< .0001	2.283	0.165	< .0001
Age at diagnosis	− 0.009	0.003	**0.0043**	0.000	0.003	0.9531	− 0.020	0.003	** < .0001**
Race—Caucasian	0.002	0.026	0.9265	− 0.038	0.026	0.1426	− 0.007	0.027	0.8045
Sex—Female	− 0.045	0.026	0.0866	0.078	0.027	**0.0033**	− 0.016	0.028	0.5567
DM Type—Type 1	− 0.031	0.037	0.3926	− 0.071	0.037	0.0570	− 0.064	0.039	0.1000
DM duration (yr)	− 0.015	0.004	**0.0002**	0.006	0.004	0.1258	− 0.023	0.004	** < .0001**
Heart Rate (bpm)	− 0.009	0.001	** < .0001**	0.001	0.001	0.3097	− 0.009	0.001	** < .0001**
Systolic BP z-score	0.009	0.015	0.5573	0.039	0.015	**0.0082**	− 0.013	0.015	0.3978
Diastolic BP z-score	− 0.010	0.017	0.5577	0.008	0.017	0.6371	− 0.002	0.018	0.9161
Days of Vigorous activity	0.006	0.006	0.2513	0.007	0.006	0.2118	0.003	0.006	0.6334
HbA1c (%)	− 0.010	0.005	0.0657	− 0.006	0.005	0.2484	0.000	0.006	0.9865
LDL-C (mg/dl)	0.000	0.000	0.3661	0.000	0.000	0.6358	− 0.001	0.000	0.0656
HDL-C (mg/dl)	0.000	0.001	0.8548	0.000	0.001	0.6698	− 0.001	0.001	0.5657
Triglycerides (mg/dl)	− 0.021	0.022	0.3475	0.026	0.022	0.2375	− 0.020	0.023	0.3911
**BMI z-score**	0.007	0.016	0.6591	0.019	0.017	0.2484	− 0.026	0.017	0.1297
**LVM index**	0.000	0.002	0.8764	0.006	0.002	**0.0001**	− 0.005	0.002	**0.0051**
**PWV carotid femoral**	− 0.046	0.011	** < .0001**	− 0.015	0.011	0.1712	− 0.028	0.011	**0.0168**
Model R^2^	0.47			0.31			0.48		

Bolded values indicate significant variables

Parameter estimates, standard error (SE) and p values from sequential linear regression models are presented above. The model presented above is the final model and included baseline covariates: age at diagnosis, race, sex, diabetes type, duration of DM, heart rate and blood pressure, physical activity, lipids, BMI, HbA1c, LVM index and carotid femoral pulse wave velocity and adjusted for clinical site. All outcomes are log transformed for modeling

We also attempted to identify clusters of participants who shared similar phenotypes that may be associated with worse diastolic function. Four phenotypes were identified as shown in Additional file 1: Table S1. Two main clusters (1 and 2) were identified first. They are largely distinguished by diabetes type, sex, race /ethnicity and traditional cardiovascular risk factors. Closer examination of the data identified Cluster 1 could actually be separated into Cluster 1A and 1B which yielded more subtle but important differences. Cluster 1A had a higher proportion of participants with type 2 diabetes (p = 0.0459) and females. In addition, individuals in Cluster 1A had higher BP z scores, higher HbA1c, and greater arterial stiffness (all p < 0.05 for Cluster 1A vs 1B except diabetes type as noted above). Closer examination of Cluster 2 identified 2A and 2B which were distinguished by diabetes type, age at diabetes diagnosis, disease duration, but also BMI and arterial stiffness. Cluster 2B had a higher proportion of participants with type 1 diabetes, shorter duration of diabetes, and they were younger with lower BMI z scores and had less arterial stiffness (all p < 0.05 for Cluster 2A vs 2B). Thus, participants in Cluster 1A had worse diastolic function as measured by all three measures of diastolic function parameters compared to those in Cluster 2B with a greater proportion in 1A having “abnormal” diastolic vs those in 2B (56.2% vs 9.5%) if using cutoffs from healthy controls.

## Discussion

LV structure and function are worse in young adults with youth-onset type 2 diabetes compared to type 1 diabetes in spite of similar diabetes duration. However, if making comparisons to an otherwise healthy control population, participants with both type 1 and type 2 diabetes have evidence of abnormal diastolic function. Traditional cardiovascular risk factors including older age at diagnosis, female sex, higher BP, heart rate, and longer diabetes duration, were all associated with worse LV diastolic function and these risk factors were observed among participants with poorer diastolic function. Diastolic dysfunction is associated with future risk of HFpEF.

Cardiovascular mortality is predicted by cardiac geometry that increases with left ventricular concentric remodeling, eccentric left ventricular hypertrophy, and concentric left ventricular hypertrophy [37, 38]. Here 25.5% of young adults with type 2 diabetes had evidence of abnormal cardiac geometry. In a large cohort of black- white teens studied in Cincinnati Ohio with type 2 diabetes studied at mean age of 18 years, abnormal cardiac geometry was seen in 20% [11]. The TODAY randomized clinical trial and its observational follow-up of young adults with type 2 diabetes found abnormal cardiac geometry in ~ 15 of the cohort at baseline [12] (mean age 18 years) and in 16% after 5 years of follow-up, respectively [35]. Older age at the time of echocardiography and differences in the cohort characteristics could explain the higher rates of abnormal geometry observed here [11, 12, 35]. Additionally, aggressive glycemic control in the TODAY clinical trial could explain the lower rates of abnormal cardiac geometry in that study.

Abnormal cardiac geometry was observed in less than < 10% of participants with type 1 diabetes and one-quarter of participants with type 2 diabetes. As abnormal geometry is virtually never seen in healthy individuals [11, 35, 39], these findings suggest risk of subclinical cardiac abnormalities in both forms of diabetes. Higher rates of abnormal cardiac geometry in type 2 diabetes are likely due to combined obesity and diabetes as cardiac remodeling is seen in obese youth without diabetes [11, 13].

Participants with type 2 diabetes had lower systolic function as measured by lower ejection fraction and worse longitudinal strain and strain rate compared to participants with type 1 diabetes, but within the range of normal for age. However, impaired ejection fraction, abnormal strain and strain rate are tied to all-cause and cardiovascular mortality long-term [40]. Diastolic function was also lower in participants with type 2 diabetes compared to type 1 diabetes even after adjustment for differences in BMI, BP and HbA1c. However, the percent with abnormal diastolic function was high in both groups (47.5% in type 1 diabetes and 58.1% in type 2 diabetes) compared to healthy controls. Given the strong link between diastolic dysfunction and HFpEF, ongoing monitoring of diastolic function and progression towards HFpEF would be valuable.

Risk factors associated with diastolic function included older age at diabetes, longer duration of diabetes, female sex, heart rate, BP, and marginally the presence of type 2 diabetes. BMI z was associated with worse diastolic function, but appeared to work indirectly through worsening LVM index or arterial stiffness at least for E/e’ and e’/a’, but may explain the worse diastolic function in the type 2 diabetes group. The presence of type 2 diabetes was marginally associated with worse diastolic function again suggesting obesity may still be an important risk factor. Cluster analysis supported these findings suggesting those with poorer diastolic function are young adult females (mid 20 s) with type 2 diabetes with duration of diabetes of more than a decade. Additionally, males with type 1 diabetes and diabetes for less than a decade may be at lower risk. Age, female sex, blood pressure, and heart rate have been associated with a greater change in diastolic function over time in young adults with type 2 diabetes in the TODAY study [35] suggesting prevention but perhaps early identification and/or more aggressive intervention of BP and heart rate may be associated with improvements in diastolic function. Additionally, those with phenotypes consistent with the highest risk category may benefit from early echocardiogram to identify and track diastolic function.

It is important to note E/A ratio, though it has been widely used as an estimator of LV diastolic function, both low and high values can be considered abnormal. In the T2D group, a low E/A ratio seemed to prevail indicating indicatinges more severe, restrictive dysfunction while higher E/A was more prevalent in the T1D group indicating more mild or moderate dysfunction. Whether these findings represent true LV diastolic dysfunction in the type 2 group or might be due to the younger age of T1D group is not known, but consistent worse values for E/e' and e'/a' in the type 2 diabetes group support the former.

Limitations of this study include absence of a recruited control group. However, published data from age similar healthy controls allowed us to quantify the degree of abnormal diastolic function in participants with diabetes. Since echocardiograms were not able to be done on all participants, only conducted at 4 of the 5 SEARCH sites, and the type 1 diabetes group was enriched with minority race/ethnic participants, it is possible our findings may not be generalizable. However, the characteristics of those getting echocardiograms vs those not getting them did not differ. The cross-sectional nature of this study precludes determination of causality, but our data identified risk factors, particularly heart rate and BP (at a single time point) that may be important for future study. Finally, sleep apnea, which is known to influence cardiac function, was not measured in this study and could have influenced the findings. Strengths of this study though include a direct comparison of a multi-ethnic population of young adults with type 1 and type 2 diabetes with similar duration, the ability to compare data to adolescents and young adults of similar age, and a large enough sample size to determine risk factors associated with diastolic function and identify clusters of individuals that may be at higher risk.

## Conclusion

In conclusion, abnormal diastolic function is seen in adolescents and young adults with both youth-onset type 1 diabetes and type 2 diabetes. In addition, 25% of young adults with type 2 diabetes had abnormal cardiac geometry at an average of 11 years of diabetes duration. These findings support monitoring cardiac structure and function in these young adults for the development of future cardiac-related complications including HFpEF and suggest aggressive interventions aimed at lowering heart rate, BP and HbA1c could be beneficial.

## Supplementary Information

**Additional file 1: Table S1.** Participant characteristics by clustering analysis.

## Data Availability

All data and materials are available upon request.

## Abbreviations

HFpEF: Heart failure with preserved ejection fraction
BP: Blood pressure
BMI: Body mass index
LV: Left ventricle
PWV: Pulse wave velocity
SD: Standard deviation
IQR: Interquartile range
LVM: Left ventricular mass
CV: Cardiovascular
