# Supplementary material for: A cross sectional study to compare cardiac structure and diastolic function in adolescents and young adults with youth-onset type 1 and type 2 diabetes: The SEARCH for Diabetes in Youth Study
Source: Cardiovasc Diabetol. 2021 Jul 7;20:136. doi: 10.1186/s12933-021-01328-0 (PMC8265135; doi:10.1186/s12933-021-01328-0)
Supplement: Supplementary file 1 — Additional file 1: Table S1. Participant characteristics by clustering analysis. [file 12933_2021_1328_MOESM1_ESM.docx]

**Additional Table S1. Participant Characteristics by Clustering Analysis**

| **Characteristics** | **1A (n=73)** | **1B (n=197)** | **2A (n=62)** | **2B (n=106)** | **p-value** |
| --- | --- | --- | --- | --- | --- |
| Diabetes Type |  |  |  |  | <.0001 |
| Type 1 | 26 (35.6%) | 97 (49.2%) | 40 (64.5%) | 84 (79.2%) |  |
| Type 2 | 47 (64.4%) | 100 (50.8%) | 22 (35.5%) | 22 (20.8%) |  |
| Age (years) | 24.5 (0.5) | 23.3 (0.3) | 27.0 (0.5) | 18.0 (0.4) | <.0001 |
| Gender |  |  |  |  | <.0001 |
| Female | 62 (84.9%) | 132 (67.0%) | 26 (41.9%) | 49 (46.2%) |  |
| Male | 11 (15.1%) | 65 (33.0%) | 36 (58.1%) | 57 (53.8%) |  |
| Race |  |  |  |  | <.0001 |
| Caucasian | 23 (31.5%) | 80 (40.6%) | 39 (62.9%) | 61 (57.5%) |  |
| Non-Caucasian | 50 (68.5%) | 117 (59.4%) | 23 (37.1%) | 45 (42.5%) |  |
| Diabetes duration (years) | 11.4 (0.3) | 10.8 (0.2) | 13.1 (0.4) | 9.1 (0.3) | <.0001 |
| Heart rate (bpm) | 90.0 (0.6) | 73.2 (0.4) | 56.5 (0.7) | 58.7 (0.5) | <.0001 |
| Systolic Blood Pressure z-score | 0.6 (0.1) | 0.0 (0.1) | -0.6 (0.1) | -0.6 (0.1) | <.0001 |
| Diastolic Blood Pressure z-score | 1.4 (0.1) | 0.9 (0.1) | 0.4 (0.1) | 0.3 (0.1) | <.0001 |
| Hemoglobin A1c (%) | 10.8 (0.3) | 9.3 (0.2) | 8.0 (0.3) | 8.6 (0.2) | <.0001 |
| BMI z-score | 1.4 (0.1) | 1.4 (0.1) | 1.3 (0.1) | 0.8 (0.1) | <.0001 |
| PWV carotid-femoral (m/sec) | 8.4 (0.2) | 7.0 (0.1) | 6.5 (0.2) | 5.5 (0.1) | <.0001 |
| **Diastolic Outcome** |  |  |  |  |  |
| E/A | 1.3 (0.1) | 1.7 (0.0) | 2.1 (0.1) | 2.1 (0.0) | <.0001 |
| E/e’ | 7.2 (0.2) | 6.9 (0.1) | 6.6 (0.2) | 6.4 (0.2) | 0.0229 |
| e’/a’ | 1.4 (0.1) | 1.7 (0.0) | 1.9 (0.1) | 2.3 (0.0) | <.0001 |

Data are unadjusted mean ± SE or n (%). p value indicates a significant difference across the four groups.
